# Movement Speed-Accuracy Trade-Off in Parkinson's Disease

**DOI:** 10.3389/fneur.2018.00897

**Published:** 2018-10-24

**Authors:** Laure Fernandez, Raoul Huys, Johann Issartel, Jean-Philippe Azulay, Alexandre Eusebio

**Affiliations:** ^1^Aix-Marseille Université, CNRS, ISM, Marseille, France; ^2^Université de Toulouse, UMR 5549 CERCO (Centre de Recherche Cerveau et Cognition), UPS, CNRS, Toulouse, France; ^3^Multisensory Motor Learning Lab, School of Health and Human Performance, Dublin City University, Dublin, Ireland; ^4^Aix-Marseille Université, APHM, CHU Timone, Department of Neurology and Movement Disorders, Marseille, France; ^5^Aix-Marseille Université, CNRS, UMR 7289, Institut de Neurosciences de la Timone, Marseille, France

**Keywords:** parkinson's disease, dopamine, speed-accuracy trade-off, rhythmicity, goal-directed movement

## Abstract

Patients with Parkinson's disease (PD) often have difficulties generating rhythmic movements, and also difficulties on movement adjustments to accuracy constraints. In the reciprocal aiming task, maintaining a high accuracy comes with the cost of diminished movement speed, whereas increasing movement speed disrupts end-point accuracy, a phenomenon well known as the speed-accuracy trade-off. The aim of this study was to examine how PD impacts speed-accuracy trade-off during rhythmic aiming movements by studying the structural kinematic movement organization and to determine the influence of dopamine replacement therapy on continuous movement speed and accuracy. Eighteen patients with advanced idiopathic Parkinson's disease performed a reciprocal aiming task, where the difficulty of the task was manipulated through target width. All patients were tested in two different sessions: ON-medication and OFF-medication state. A control group composed of healthy age-matched participants was also included in the study. The following variables were used for the analyses: Movement time, Error rate, effective target width, and Performance Index. Percentage of acceleration time and percentage of non-linearity were completed with kinematics patterns description using Rayleigh-Duffing model. Both groups traded off speed against accuracy as the constraints pertaining to the latter increased. The trade-off was more pronounced with the PD patients. Dopamine therapy allowed the PD patients to move faster, but at the cost of movement accuracy. Surprisingly, the structural kinematic organization did not differ across group nor across medication condition. These results suggest that PD patients, when involved in a reciprocal aiming task, are able to produce rhythmic movements. PD patients' overall slowing down seems to reflect a global adaptation to the disease in the absence of a structurally altered kinematic organization.

## Introduction

Parkinson's Disease (PD) is clinically characterized by the parkinsonian triad (bradykinesia, rigidity and rest tremor) and patients often have difficulties in generating rhythmic movements (1). In daily life, this may lead to impairments in various activities such as walking, writing and manipulating objects. Such skills, by definition, require movement adjustments to deal with the prevailing accuracy constraints. Succeeding in these tasks comes with the cost of diminished movement speed, whereas increasing movement speed disrupts end-point accuracy.

**Figure 1:**
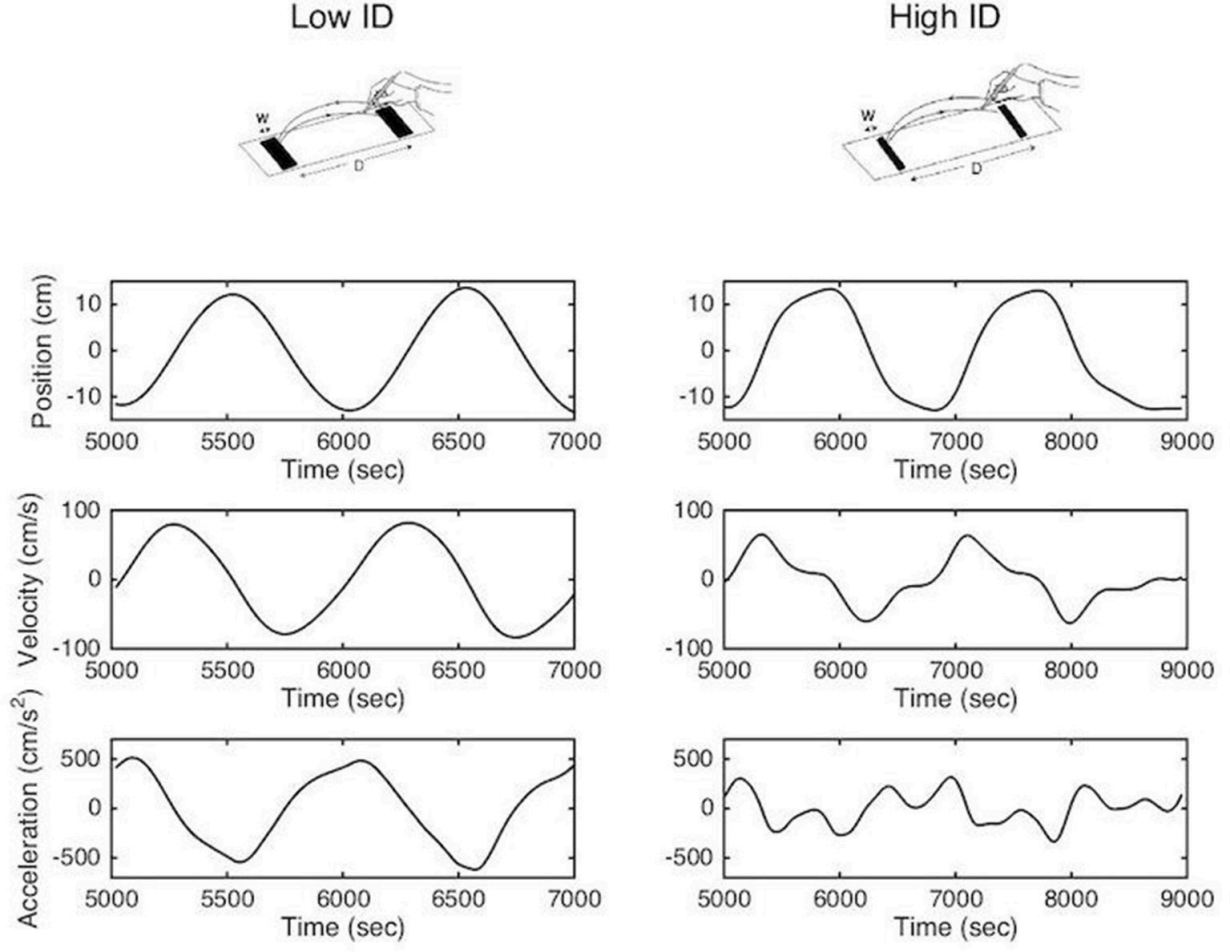
Methodological representation of reciprocal aiming task under low (upper left panel) and high (upper right panel) index of difficulty. Position, velocity and acceleration profiles are represented for two successive aiming cycles.

This trade-off between movement speed and accuracy is formalized in Fitts' law, which states that movement time relates linearly with the index of difficulty (ID), which quantifies task difficulty, in aiming tasks (2–4). In the reciprocal paradigm, the analysis of the kinematic patterns of movements during the task have revealed two different organizations (5–7). When the accuracy constraints are low the movement can be defined as purely rhythmic; smooth bell-shaped velocity profiles, with symmetrical acceleration and deceleration components, are observed and the deceleration and re-acceleration phases are fully merged (8, 9). When accuracy constraints are high the movement tends to become a concatenation of discrete movements. Velocity profiles become increasingly asymmetric mainly due to an elongated deceleration phase and acceleration tends to zero at movement reversal point (10) (see Figure 1). This slowing down has been attributed to on-line sensorimotor integration during the movement's final phase (11–14), potentially corrective in nature (15). In addition to the topographic analysis, Mottet and Bootsma (10) have modeled the kinematic movement patterns as a function of task difficulty in terms of a nonlinear oscillator that captures the structural movement organization assembled within the neuro-motor system. They showed that the gradual scaling of ID resulted in a gradual change of the model parameters but that the model terms were invariant to the ID scaling. These results have been confirmed numerous times since in healthy subjects (16, 17).

To date, how PD patients deal with the speed-accuracy trade-off has been mostly investigated for single aiming movements with the discrete Fitts' task (18–21). Few studies have investigated the kinematic patterns of continuous aiming movements. During a continuous side-to-side zig-zag pattern, (22) found that PD patients perform less accurately than controls when movement speed is fixed. In a continuous task (23) observed similar results in PD patients' movement accuracy when the movement speed was unconstrained. Dopamine replacement therapy (DRT) was found to increase movement speed but not accuracy (23). However, these studies only investigated macroscopic movement variables such as movement speed and accuracy. Despite the well-known PD patients' difficulties in generating rhythmic movements such as finger taps or walking, none of these authors have investigated PD patients' capabilities to produce accurate rhythmic movements. Here, we study the structural kinematic movement organization in a reciprocal aiming task performed by PD patients both OFF and ON their dopaminergic medication and compare that to a healthy subjects group.

The goal is to study if and how PD alters the structural kinematic movement organization when accuracy constraints are manipulated, and to determine if and how DRT affects this kinematic organization as well as the movement speed and accuracy of PD patients. Our hypothesis was that PD patients' reciprocal aiming movements are slower, more non-linear (i.e., built on several acceleration bursts) and performed in a more discrete fashion across the range of task difficulties tested compared to matched-control participants.

## Materials and methods

### Participants

Eighteen patients with advanced idiopathic Parkinson's disease (10 females and 5 males, mean age 60.1 years, SD 9.1 years, mean disease duration 11.8 years, SD 6.6 years) participated after signing an informed consent. This study was carried out in accordance with the recommendations of Comité de Protection des Personnes Sud Méditerranée with written informed consent from all subjects. All subjects gave written informed consent in accordance with the Declaration of Helsinki (clinical details of all patients in Table 1). The protocol was approved by the Comité de Protection des Personnes Sud Méditerranée. All patients were right-handed and had normal or corrected-to-normal vision. None of the patients had cognitive impairment (Mattis Dementia Rating Scale ≥130) nor visuospatial impairment; indeed all patients, although with advanced PD, were potential candidates for deep brain stimulation surgery. In addition, as we were primarily interested in studying akinesia and to avoid any bias due to confounding movements, we did not include patients with severe tremor (≥3 for items 20 and/or 21 of the UPDRS III) or severe dyskinesias (>7 at the Marconi dyskinesia scale). Eighteen right-handed healthy age-matched control participants (13 females and 5 males, mean age 59.39 years, SD 8.37 years) without any known motor disorders also participated in this study.

**Table 1:** Demographic and drug details of PD Patients and controls.

**No**.	**Sex**	**Age**	**Years (Diagnosis)**	**Medication**	**UPDRS III OFF**	**UPDRS III ON**	**AIMS OFF**	**AIMS ON**	**Tremor OFF**	**Tremor ON**
1	F	56	10	l-dopa 550 mg; entacapone 1,200 mg; pramipexole ER 2.10 mg; rasagiline 1 mg	17	2	0	0	0	0
2	F	69	30	l-dopa 350 mg; pramipexole 2.1 mg	24	9	0	5	0	1
3	F	59	8	l-dopa 700 mg; pramipexole ER 2.62 mg; entacapone 1,200 mg	30	10	0	1	1	0
4	M	57	14	l-dopa 1,000 mg; entacapone 800 mg; ropinirole ER 16 mg	31	12	0	3	0	0
5	M	65	7	l-dopa 1,300 mg; ropinirole ER 4 mg	48	9	0	6	1	0
6	F	70	10	l-dopa 600 mg	21	5	0	1	0	0
7	M	69	11	l-dopa 750 mg; rasagiline 1 mg	18	6	0	7	1	0
8	F	65	11	l-dopa 400 mg; entacapone 800 mg; ropinirole ER 10 mg	25	14	2	2	0	0
9	F	68	6	l-dopa 675 mg; rasagiline 1 mg	36	23	0	0	0	1
10	M	46	6	l-dopa 475 mg; entacapone 1,000 mg; ropinirole ER 24 mg; rasagiline 1 mg	21	1	2	7	1	0
11	M	63	16	l-dopa 900 mg; entacapone 1,000 mg; ropinirole ER 16 mg	33	14	7	4	0	0
12	F	68	10	l-dopa 625 mg; entacapone 1,000 mg; rotigotine 8 mg; rasagiline 1 mg	26	15	0	6	0	0
13	M	41	5	l-dopa 850; entacapone 1,200 mg; pramipexole ER 0.52 mg; rasagiline 1 mg	31	1	0	7	1	0
14	F	56	8	l-dopa 500 mg; ropinirole ER 6 mg; rasagiline 1 mg	15	3	1	4	0	0
15	F	44	8	l-dopa 675 mg; entacapone 1,200 mg	23	3	2	4	1	0
16	F	56	22	l-dopa 150 mg; entacapone 800 mg; amantadine 100 mg	21	3	3	4	0	0
17	M	70	10	l-dopa 750 mg; entacapone 1,200 mg; rasagiline 1 mg; pramipexole ER 1.05 mg	10	0	3	3	0	0
18	M	59	21	l-dopa 1,000 mg; entacapone 800 mg; bromocriptine 7.5 mg	38	18	0	2	0	0
**PATIENTS**										
8M	Mean	60.06	11.8		26	8.22	1.11	3.67	0.33	0.11
10F	SD	9.14	6.6		9.25	6.66	1.84	2.35	0.49	0.32
*T*-test					*p* < 0.001		*p* < 0.005			
**CONTROLS**										
5M	Mean	59.39								
13F	*SD*	8.37								

*UPDRS = Unified Parkinson's Disease Rating Scale motor*.

*Dyskinesia = Severity of dyskinesias assessed using the Marconi dyskinesia Scale*.

*Post action Tremor score for preferred hand*.

### Apparatus and task

Participants were seated comfortably at a table, facing a laptop screen (Dell Vostro 1720, 1,440 × 900 pixels) with a connected graphics tablet (Wacom Ultra Pad A3) placed horizontally on the table in front of them. Left-right motion of a hand-held, non-marking stylus on the graphics tablet was linked to the left-right displacement of a cursor on the computer screen via a dedicated software program developed in the laboratory. The task was to move the cursor back and forth between two targets depicted on the screen (i.e., Fitts task). The target was a rectangle of a given width (depending on the ID) with a height corresponding to the height of the screen. The gain between the displacement of the stylus on the graphics tablet and the displacement of the cursor on the computer screen was unitary (i.e., 1 cm on the graphics tablet corresponded to 1 cm on the computer screen). Movement was recorded along the horizontal and vertical direction; the latter was not further analyzed. The position of the stylus on the graphics tablet was sampled at a frequency of 150 Hz.

### Recordings and procedure

The experiment consisted of two sessions: either after overnight withdrawal of the antiparkinsonian medication (hereafter referred to as OFF-dopa condition) or after administration of their morning levodopa-equivalent dose of medication (ON-dopa condition). The order of the sessions was inverted with six patients starting with the ON-dopa session. The time elapsed by the two sessions in total was about 1 h. UPDRS motor scoring was performed in both OFF- and ON-dopa conditions to ensure that the medication induced a correct ON-dopa state (26 ± 9.3 vs. 8.2 ± 6.7, respectively; paired sample *t*-test: *t* = 10.46, ddl = 17, *p* < 0.001) (Table 1). 11 patients were taking extended release dopamine agonists which were not discontinued 5 half-lives before the experiment but only 24 h; some residual effect of dopamine agonists may thus have been present in the OFF condition for these patients. The participants from the control group also performed two identical sessions (without medication) in a row in order to guarantee the same number of experimental conditions between the two groups.

All participants performed a reciprocal Fitts' task as described above. The instructions were to perform the task as fast and as accurately as possible. Errors were defined as movement reversals occurring outside the target area. A trial consisted of 50 consecutive aiming movements from one target to the other (i.e., 25 cycles). Participants performed three levels of task difficulty: *ID* = 3, 4, and 5 with *ID* = log_2_ (2*D*/*W*) (2). The 20 cm inter-target distance *D* (from center to center) was fixed across all conditions. The target size *W* was equal to 6.50, 3.25, and 1.62 cm for *ID* 3, 4, and 5, respectively. A familiarization phase was included at the beginning of both sessions. Each of the two sessions was composed of three blocks. In each block, the three *ID* conditions were presented in a randomized order for a total of nine experimental trials. A short rest period was provided between each condition and a longer break between the blocks. The 50 aiming movements had to be performed with fewer than 20% errors. If this criterion was not met, the trial was re-run. The first two aimings were not analyzed to avoid transient behavior in the analysis.

### Data analysis

The position time series were filtered with a dual-pass, second-order Butterworth filter, with a cut-off frequency of 8 Hz. Velocity and acceleration were subsequently derived using a 3-point central difference technique. The analysis focused on movement time, error rate, effective target width, performance index, percentage of acceleration time, non-linearity percentage as well as descriptions of the kinematics patterns produced. For each session, measures were averaged across the three trials for each of the three conditions. Individual movements were obtained by identifying their reversals (i.e., position extremes). For each trial, movement time (*MT*) was defined as the mean half cycle time, from one movement extremum to the next. The error rate was defined as the number of movement reversal outside targets limits. The effective width of the target (*W*_*e*_) was defined as 1.96 times the standard deviation of the actual end-point distribution at movement reversal Welford (24). Relative *W*_*e*_ was obtained as the ratio *W*_*e*_/*W*, where *W* is the actual width of the corresponding target.

The Index of Performance (*IP*) was computed from MT and *W*_*e*_ according to the International Organization for Standardization (25) as the following: IP = IDe/MT, where IDe is the effective index of difficulty adjusted by using effective width of the target computed as: IDe = log2 (D/ *W*_*e*_ +1).

The percentage of acceleration time was defined as the time to peak velocity divided by the movement time. The kinematics patterns were first analyzed by portraying the Hooke portrait from individual aiming cycles representing the entire acceleration signal as a function of the entire signal of position (10). To keep the acceleration scale comparable across trials without changing the portrait shape, the data were normalized as a function of MT and amplitude (26). To this end, time was rewritten in units of cycle time and, amplitude rewritten in units of amplitude/2. In this normalized space, simple harmonic movement yields a straight line with a negative unit slope in the Hooke plane. Any deviation thereof reflects non-linearities in the dynamics underlying movement (10). The normalization procedure provides the possibility to quantify these changes through extraction of the non-linearity percentage (NL). From the Hooke's portraits the *R*^2^ of the linear regression of position onto acceleration was used to determine the amount of variance that can be attributed to purely harmonic motion. The residue of this regression measures the influence of the non-linear component using 1–*R*^2^ = NL (10).

The Rayleigh-Duffing model (RD model) (10) was fitted to the average normalized cycle of each participant under each experimental condition using multiple linear regression of acceleration onto linear and cubic position and velocity (see Equation 1) providing a goodness of fit measure (coefficient of determinacy *R*^2^), as well as specification of each of the four model coefficients. The RD model reads:

(1) $$\ddot{x}+C_{10}x-C_{30}x^{3}-C_{01}\dot{x}+C_{03}{\dot{x}}^{3}=0$$

### Statistical analysis

Repeated-measures ANOVAs were performed between groups (PD patients, control), sessions (Session 1, Session 2) and task difficulty (ID 3, ID 4, and ID 5) as factors. Sphericity was assessed for each dependent variable and the Greenhouse–Geisser's correction was applied when sphericity was not met. *Post hoc* analysis using Bonferroni's correction was used in order to detail significant effects. Statistical significance was set a *p* < 0.05.

Differences in MT, Error rate as well as *W*_*e*_ /W ratio, between treatment sessions were assessed using analysis of covariance (ANCOVA) with the clinical data of Dyskinesia score (Marconi) as well as with the post action tremor score as a covariate to test whether the significant changes of the dependent variables were due to dyskinesias or to tremor.

## Results

### Movement time

The regression analysis using *MT* data from the PD patients all well as those from the control group indicated a linear relationship between *MT* and ID (with *R*^2^ > 0.99 for all regressions). For both groups, *MT* increased linearly the ID, with slopes of 0.346 and 0.394 for the ON-dopa and OFF-dopa sessions for the PD group, respectively, and slopes of 0.262 and 0.258 for session 1 and 2 respectively, for the Control group. The slope was significant different across Group [*F*_(1, 34)_ = 9.39, *p* = 0.004, η_2_ = 0.87] whereas neither the effect of Session nor the interaction Group X Session were significant.

**Figure 2:**
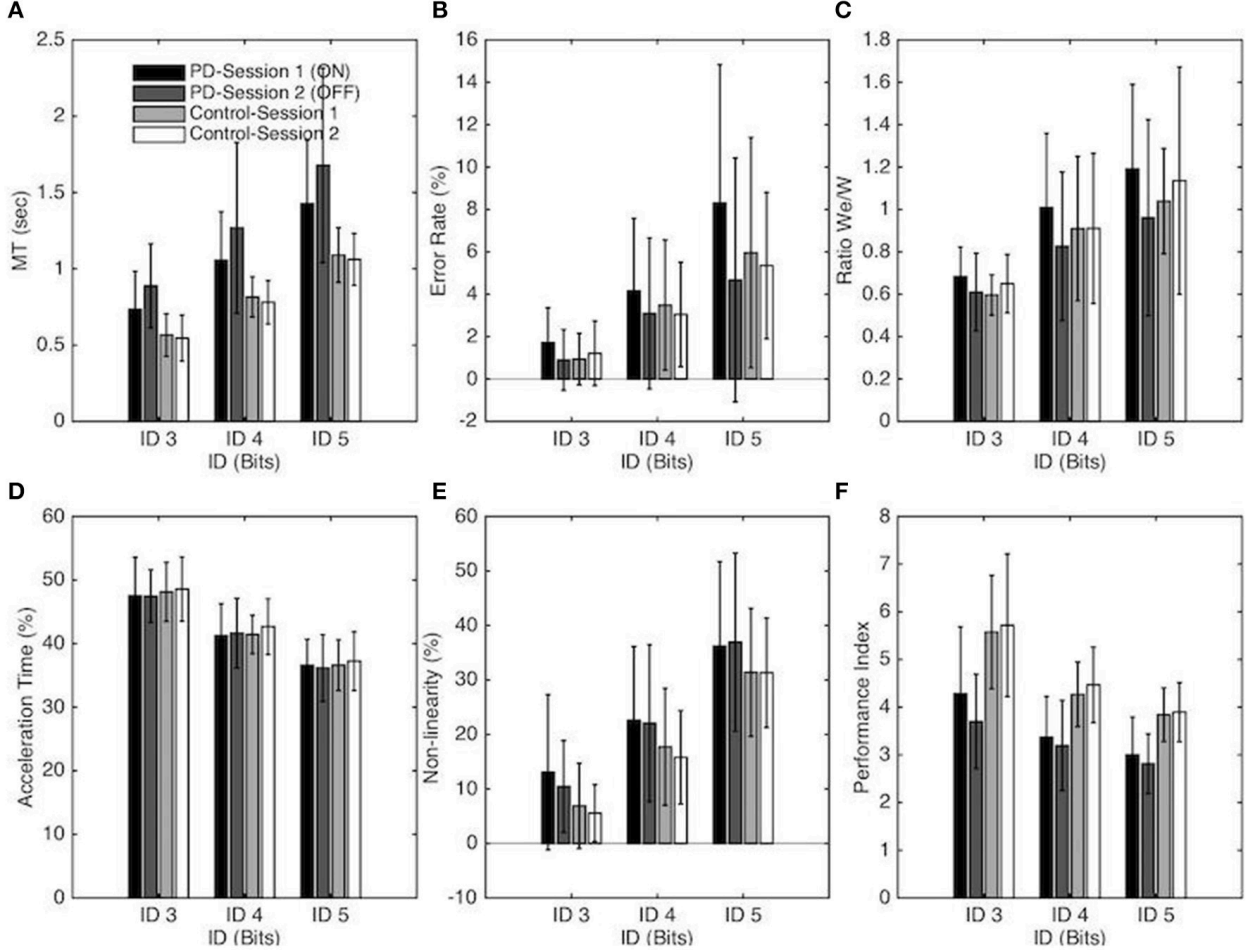
Each panel depicts one performance variable as a function of the Index of difficulty, Group, and session for the PD patients and control groups. In panel **A-F** Session 1 and Session 2 correspond to the ON and OFF conditions for the PD patients group, respectively. The vertical bars depict the standard deviation of the mean. Each panel represents the following performance variable: **(A)** Average movement time, **(B)** Average error rate percentage, **(C)** We/W ratio, **(D)** Percentage of Acceleration time, **(E)** Non-linearity percentage and **(F)** Index of Performance.

A significant main effect of ID on *MT* [*F*_(1, 18, 40.2)_ = 233.61, *p* < 0.001, η_2_ = 0.87] confirmed that *MT* increased with task difficulty. A significant main effect of Group [*F*_(1, 34)_ = 16.69, *p* < 0.001, η_2_ = 0.33] demonstrated that PD patients moved slower than control participants (on average *MT* = 0.81s ± 0.23 for the Control group and *MT* = 1.17s ± 0.35 for the PD patients). The interaction between Group and Session was found to be significant [*F*_(1, 34)_ = 5.77, *p* = 0.022, η_2_ = 0.15]; a simple effects analysis revealed that PD patients moved slower (*p* < 0.001) without medication (1.28s ± 0.39) than with medication (1.07s ± 0.35) whereas *MT* between sessions was not significantly different for the control group. The interaction between task difficulty and group was also significant [*F*_(1, 18)_ = 7.15, *p* < 0.002, η_2_ = 0.17]. As evident from the regression analysis (see above), the *MT* increase as a function of ID was stronger for PD patients than for the control group. The simple effect decomposition indicated that *MT* for each ID was significantly different from all others for the PD group (*p* < 0.001) as well as for the control group (*p* < 0.001) (see Figure 2A).

Considering the dyskinesia score (MARCONI) for the PD group, the ANCOVA demonstrated that the slopes of the relation between the dyskinesia score and MT were not significantly different for the two sessions conditions (i.e., ON-dopa and OFF-dopa sessions for the PD group) [*F*_(1, 32)_ = 1.82, *p* = 0.19] (see Table 2).

**Table 2:** Results from the ANCOVA performed on MT, Error rate and We/W ratio to test the interaction of (i) Dopamine treatment and dyskinesia score (Marconi), and (ii) Dopamine treatment and tremor score post action.

	***ANCOVA, F/p value***	***Eta-square***
**MOVEMENT TIME**		
Dopa Session (ON/OFF)	0.46/0.50	0.013
AIMS Score	1.46/0.23	0.041
Session*AIMS	1.82/0.19	0.051
Dopa Session (ON/OFF)	1.07/0.31	0.029
Tremor score	3.12/0.09	0.085
Session*Tremor	0.71/0.41	0.019
**ERROR RATE**		
Dopa Session (ON/OFF)	0.79/0.381	0.022
AIMS Score	0.98/0.329	0.027
Session*AIMS	1.93/0.174	0.054
Dopa Session (ON/OFF)	3.33/0.07	0.077
Tremor score	0.16/0.70	0.004
Session*Tremor	7.98/0.01	0.183
**WE/W RATIO**		
Dopa Session (ON/OFF)	3.3/0.07	0.092
AIMS Score	0.07/0.79	0.002
SessionAIMS	0.29/0.59	0.008
Dopa Session (ON/OFF)	5.34/0.03	0.103
Tremor score	0.01/0.90	0.0002
Session*Tremor	14.63/0.001	0.281

Considering the post action tremor score for the PD group, the ANCOVA demonstrated that the slopes of the relation between tremor score and MT ratio were not significantly different for the two sessions conditions (i.e., ON-dopa and OFF-dopa sessions for the PD group) [*F*_(1, 32)_ = 0.71, *p* = 0.19] (see Table 2).

### Error rate

The *Error Rate* also increased significantly with ID [*F*_(1, 35, 45.88)_ = 37.81, *p* < 0.001, η_2_ = 0.53, ER = 1.19, 3.45, and 6.07 for ID 3, 4, and 5, respectively]. The *Error Rate* (ER) was significantly affected by Session [*F*_(1, 34)_ = 4.95, *p* = 0.033, η_2_ = 0.13]; on average more errors were made in Session 1 (4.10%) than in Session 2 (3.04%). *Post-hoc* analysis demonstrated that this difference was significant for the PD group (*p* = 0.009) but not for the control group. On average, the patients missed the targets more often in the ON-Dopa condition (4.7%) compared to the OFF-Dopa condition (2.9%).

The decomposition of these main effects showed that the *Error Rate* for each ID was significantly different from all others for the PD group (*p* < 0.001) as well as for the control group (*p* < 0.01). Neither the main effect of Group nor the interaction effect reached significance (see Figure 2B).

Considering the clinical data through the dyskinesia score (MARCONI) for the PD group, the ANCOVA demonstrated that the slopes of the relation between dyskinesia score and Error Rate were not significantly different for the two sessions conditions (i.e., ON-dopa and OFF-Dopa sessions for the PD group) [*F*_(1, 32)_ = 1.93, *p* = 0.17) (see Table 2).

Considering the post action tremor score for the PD group, the ANCOVA demonstrated that the slopes of the relation between tremor score and Error Rate were significantly different for the two sessions conditions (i.e., ON-dopa and OFF-dopa sessions for the PD group) [F_(1, 32)_ = 7.98, *p* < 0.01) (see Table 2).

### We/W ratio

The repeated-measures ANOVA revealed a significant main effect of ID on *W*_*e*_*/W ratio* [*F*_(1, 95, 66.43)_ = 33.11, *p* < 0.001, η_2_ = 0.49], with a higher ratio for higher ID (on average *We/W* = 0.63, 0.91, and 1.08, for ID 3, 4, and 5 respectively). For the PD group, decomposition of this main effect demonstrated that the *We/W ratio* was significantly different for each ID (*p* < 0.01). For the Control group, the difference on *We/W ratio* was significant between ID 3 and ID 4 (*p* < 0.001) and between ID 3 and ID 5 (*p* < 0.001) but not between ID 4 and 5. The interaction between Session and Group was also found to be significant [*F*_(1, 34)_ = 7.22, *p* = 0.011, η_2_ = 0.18]. A simple effects analysis revealed that the ratio was higher for the ON-Dopa conditions (0.96 ± 0.26) in comparison with the OFF-Dopa conditions (0.80 ± 0.18) for the PD group (*p* < 0.01) but not for the control group (see Figure 2C).

Considering the dyskinesia score (MARCONI) for the PD group, the ANCOVA demonstrated that the slopes of the relation between dyskinesia score and We/W ratio were not significantly different for the two sessions conditions (i.e., ON-dopa and OFF-dopa sessions for the PD group) [*F*_(1, 32)_ = 0.29, *p* = 0.59] (see Table 2).

Considering the post action tremor score for the PD group, the ANCOVA demonstrated that the slopes of the relation between tremor score and We/W ratio were significantly different for the two sessions conditions (i.e., ON-dopa and OFF-dopa sessions for the PD group) [*F*_(1, 32)_ = 14.63, *p* < 0.001] (see Table 2).

### Index of performance

The *Performance Index* (*IP*) changed significantly with ID [*F*_(1, 24, 62.33)_ = 98.51, *p* < 0.001, η_2_ = 0.74] with a decreasing *IP* with higher ID (on average IP = 4.82, 3.82, and 3.39, for ID 3, 4, and 5, respectively). The decomposition of this main effect showed that the *IP* for each ID was significantly different from all others for the PD group (*p* < 0.001) as well as for the control group (*p* < 0.01). The *IP* also differed significantly for the two groups [*F*_(1, 34)_ = 21.65, *p* < 0.001, η_2_ = 0.39] with a higher *IP* for the Control group in comparison with the PD group (on average *IP* = 4.63 ± 0.82, and 3.39 ± 0.53, for Control group and PD group respectively). The interaction between Session and Group was also found to be significant [*F*_(1, 34)_ = 4.4, *p* = 0.044, η_2_ = 0.11]. A simple effects analysis revealed that *IP* was higher for the ON-Dopa conditions (3.55 ± 0.66) in comparison with the OFF-Dopa conditions (3.23 ± 0.44) for the PD group (*p* < 0.05) but not for the control group (see Figure 2F).

### Percentage of acceleration time (%AT)

The (relative) acceleration time changed significantly with ID [*F*_(1, 43, 48.74)_ = 209.94, *p* < 0.001, η_2_ = 0.86), with a decreasing *%AT* with higher ID (on average *%AT* = 47.92 ± 0.53, 41.75 ± 0.64, and 36.65 ± 0.45%, for ID 3, 4, and 5, respectively). Decomposition of this main effect demonstrated that *%AT* was significantly different from all others ID for the PD group (*p* < 0.001) and the Control group (*p* < 0.001). Neither the main effects of Session, Group or the interaction effect were significant (see Figure 2D).

### Non-linearity (NL)

The repeated-measures ANOVA revealed a significant main effect of ID on the *Non-linearity* [*F*_(1, 34, 45.58)_ = 318.03, *p* < 0.001, η_2_ = 0.90] with an increasing *NL* with higher ID (on average *NL* = 8.99, 19.55, and 33.96%, for ID 3, 4, and 5, respectively). Decomposition of this main effect demonstrated that *NL* was significantly different from all others ID for the PD group (*p* < 0.001) as well as for the Control group (*p* < 0.001). The main effects of Session and Group or the interaction effects were not significant (see Figure 2E).

### Kinematics patterns

**Figure 3:**
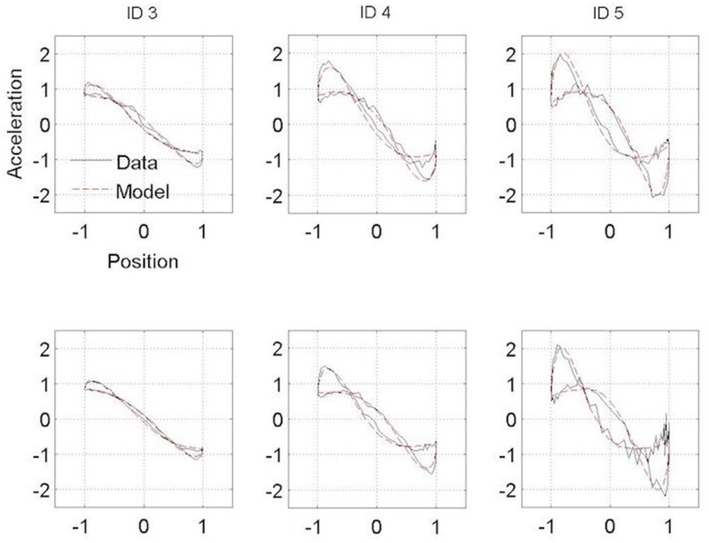
Normalized averaged Hooke portraits of the movements observed under the ON-medication session (upper panel) and OFF-medication session (lower panel) for the three ID for PD patients group. The black line represents the Hooke portraits experimental data and the red dotted line the Hooke portraits from the RD model.

**Figure 4:**
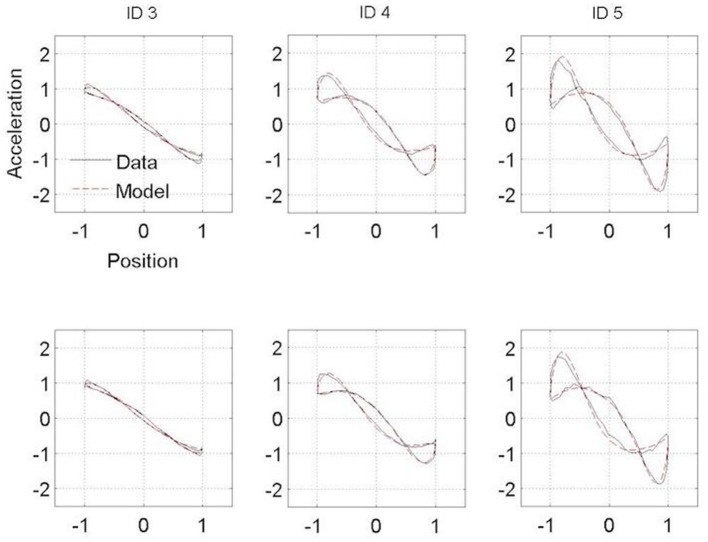
Normalized averaged Hooke portraits of the movements observed under the Session 1 (upper panel) and Session 2 (lower panel) for the three ID for control group. The black line represents the Hooke portraits experimental data and the red dotted line the Hooke portraits from the RD model.

Figures 3, 4 present the Hooke portraits (acceleration vs. position) for all experimental conditions. For both groups, low levels of difficulty (i.e., ID 3) gave rise to almost perfectly linear Hooke portraits (Figures 3, 4, left panels). For both PD patients and controls, when task difficulty increased, the Hooke portraits increasingly deviated from a straight line to an N-shaped curve observed for ID = 4 and ID = 5. Hooke portraits became asymmetric, with a deceleration phase longer than the acceleration phase. At last, acceleration was closer to zero at the vicinity of the target under ID 5 for both groups, indicating that the participants almost stopped upon arrival in the targets. Overall, the qualitative characteristics and global aspect of kinematics patterns (i.e., Hooke portraits) were identical for both groups whereas local irregularities and more jagged trajectories were observed for the PD patients group but not for control group.

### Model fitting

The RD model adequately captured the range of behavioral patterns observed, explaining 94.44 % of the variance on average. The repeated-measures ANOVA on the coefficient of regression (*R*^2^) indicated a significant main effect of ID [*F*_(1, 14, 38.83)_ = 19.41, *p* < 0.001, η_2_ = 0.36] with bests fits were obtained for low ID (on average *R*^2^ = 0.97, 0.95, and 0.91 for ID 3, ID 4, and ID 5, respectively). Decomposition of this main effect demonstrated that *R*^2^ was significantly different across all IDs for the PD group (*p* < 0.01) (on average *R*^2^ = 0.96, 0.93, and 0.87 for ID 3, ID 4, and ID 5, respectively) whereas no significant differences were obtained for the control group (on average *R*^2^ = 0.99, 0.98, and 0.94 for ID 3, ID 4, and ID 5, respectively).

A significant main effect of Group [*F*_(1, 34)_ = 4.9, *p* < 0.034, η_2_ = 0.13) also indicated that better fits were obtained for the control group than the PD group (*R*^2^ = 0.97 and 0.92, respectively).

#### Linear (C_10_) and non-linear (C_30_) stiffness

**Figure 5:**
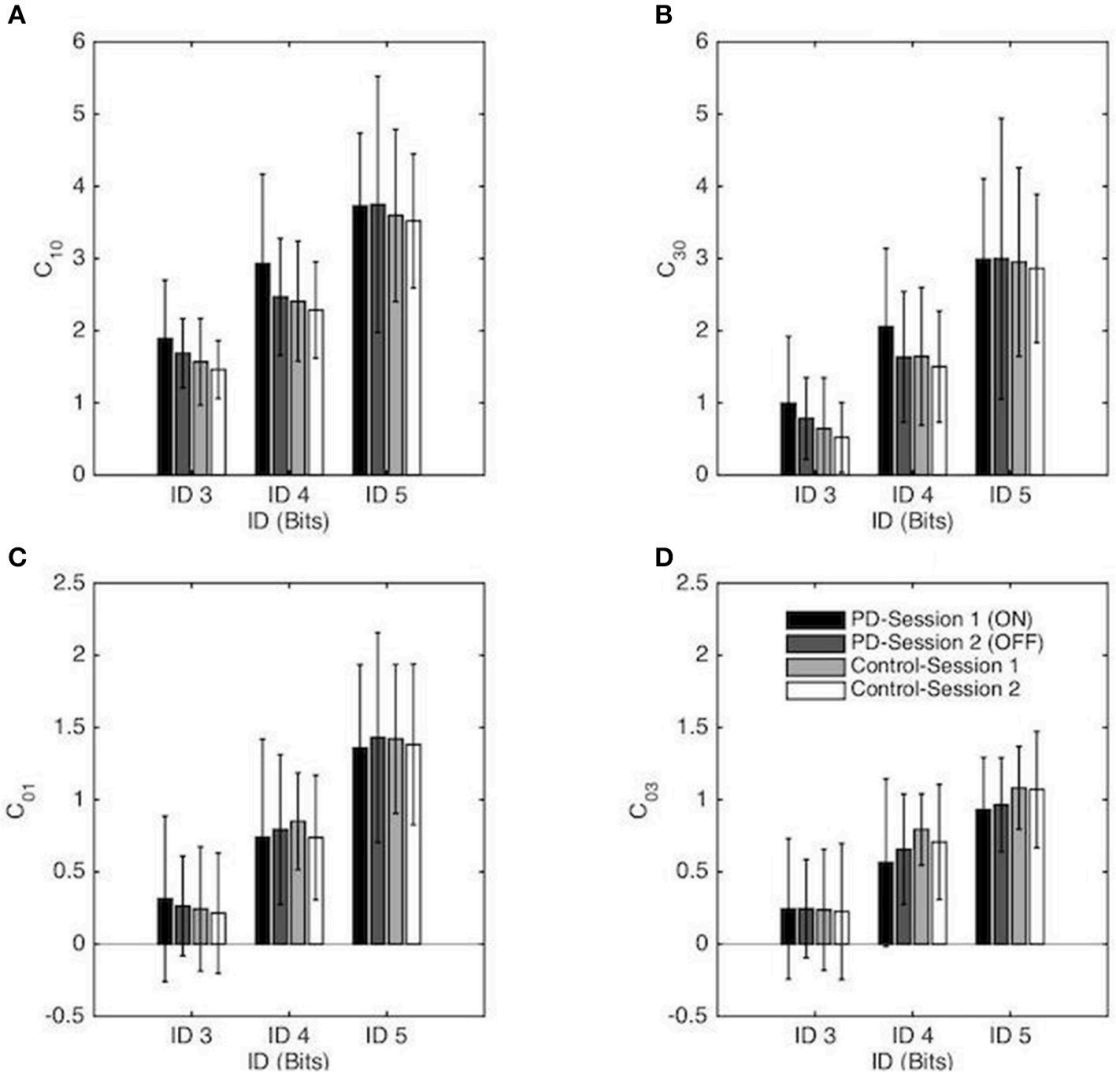
Coefficients of the RD model (normalized in space and time) as a function of the Index of Difficulty for both sessions for the PD patients group and the control group. Session 1 and Session 2 correspond, respectively, to the ON and OFF conditions for the PD patients group. The vertical bars depict the standard deviation of the mean. Linear and non-linear coefficients of position-dependent (conservative) terms are presented in **(A,B)**, respectively, and linear and non-linear coefficients of the velocity-dependent terms are presented in **(C,D)**, respectively.

Repeated-measures ANOVA revealed a significant main effect of ID for the linear stiffness term (*C*_10_) [*F*_(1, 55, 52.67)_ = 155.95, *p* < 0.001, η_2_ = 0.82], with a higher linear stiffness for higher ID (on average *C*_10_ = 1.65, 2.52, and 3.65 for ID 3, 4 and 5 respectively) as well as the non-linear stiffness term (*C*_30_) [*F*_(1, 35, 46.03)_ = 193.38, *p* < 0.001, η_2_ = 0.85] with higher non-linear stiffness for higher ID (on average *C*_30_ = 0.74, 1.71, and 2.95 for ID 3, 4 and 5 respectively). Decomposition of these mains effect demonstrated that both *C*_10_ and *C*_30_ were significantly different from all others ID for the PD group and for the Control group (all *p* < 0.001). No other effects were significant (see Figures 5A,B).

#### Linear (C_01_) and non-linear (C_03_) damping

Repeated-measures ANOVA revealed a significant main effect of ID for the linear damping term (*C*_01_) [*F*_(1, 41, 47.83)_ = 173.59, *p* < 0.001, η_2_ = 0.84), with a higher linear damping for higher ID (on average *C*_01_ = 0.26, 0.78, and 1.40 for ID 3, 4, and 5 respectively) as well as the non-linear damping term (*C*_03_) [*F*_(1, 36, 46.31)_ = 106.8, *p* < 0.001, η_2_ = 0.76] with higher non-linear damping for higher ID (on average *C*_03_ = 0.24, 0.68, and 1.01 for ID 3, 4 and 5 respectively). Decomposition of these mains effect demonstrated that both *C*_01_ and *C*_03_ were significantly different from all others IDs for the PD group and for the Control group (all *p* < 0.001). No other effects were significant (see Figures 5C,D).

## Discussion

The key findings of this study are that (i) the speed-accuracy trade-off is at work in patients with moderate PD in reciprocal precision aiming extending the findings previously obtained in discrete tasks, (ii) dopaminergic medication leads PD patients to favor speed over accuracy, (iii) the movement organization of rhythmic movements is structurally similar between PD and controls.

### Speed-accuracy trade-off in PD patients

The MT for both groups (i.e., PD and healthy group) was in line with Fitts' law; i.e., movement time increased linearly with increasing task difficulty. MT results of the current study corroborated previous results obtained when PD patients faced accuracy constraints in discrete aiming task (21, 27). As expected, PD patients were slower than healthy subjects. In addition, we found that PD patients were proportionally slower with increasing task difficulty than the control participants which could be argued as a lower information capacity of the motor system (2) as suggested by the information theory.

Since PD patients OFF medication moved slower than the participants in the control group, one could expect PD patients' movement to be more accurate. The local variability (i.e., end-point variability of movement, We/W ratio) is known to be positively correlated with movement speed for healthy participants (28, 29). We found that this relation held with PD. However, neither the error rate nor the We/W ratio differed between groups. Further, the evolution of those variables with increasing ID did not affect PD patients differently than healthy controls. The results highlight that local variability for all levels of accuracy constraints is not affected by PD medication.

Previous studies have demonstrated that in a tapping task, speed and amplitude of repetitive finger movement were lower in PD patients than healthy patients (30). In the case of a discrete aiming task, studies revealed that PD patients' movement were faster (with higher peak velocity and acceleration) in a no-target condition compared with a target condition and significantly lower than those of controls (31). The target condition forced PD patients to slow down more than healthy controls, and to adjust their movements according to the accuracy requirement (21, 32, 33). We demonstrated that the slowing down of the PD patients' reciprocal movements most likely served to insure that end-point control matched the imposed accuracy constraints. In addition, it has been shown that when PD patients were trained to move at the same speed as controls while producing continuous movements (a zigzag pattern), they showed significantly more spread of movement end-points, suggesting that PD patients were able to increase their movement speed to match control participants' preferred speed but at the expense of accuracy (22). Our results corroborate previous insights, and expand it to continuous rhythmic movements in showing that PD does not affect movement end-point accuracy, but that the conservation of accuracy comes at the cost of slowing down. Overall, this leads to an impaired performance as measured by the index of performance (IP), which was found to be lower for PD patients than controls. These results are compatible with a scheme where PD patients OFF medication, asked to move as fast and accurately as possible, implicitly chose to move slower to maintain the accuracy. These results obtained in a rhythmic task extend previous findings suggesting that bradykinesia could be interpreted as a compensatory response to match accuracy constraints, that is to say, compensation for a primary impairment of the speed-accuracy trade-off (22, 34).

### Dopamine treatment and accuracy control

In line with the literature we found that movement speed increases when PD patients receive dopamine treatment (23, 35–38). However, this increase in speed was detrimental to the endpoint accuracy: since both the error rate and We/W ratio increased after levodopa administration, in addition, we found that this impaired accuracy was not caused by levodopa induced dyskinesias whereas the post action tremor score in average decreased after levodopa administration.

Taken together, these results show that dopamine treatment causes PD patients to trade accuracy for speed relative to their performance OFF medication. Relevant in this regard is the observation that dopamine therapy affects action impulsivity, and in particular reduces the ability to abort and suppress muscle activation (39). Similarly, in a decision making task using a modified version of the moving dots paradigm, it was shown that levodopa increases the number of errors compared to OFF levodopa when the instruction is “as fast and accurately as possible” but does not so when the instructions favor speed over accuracy (40) It should be stressed that, in our task the instructions did not vary; participants were always required to execute the task “as fast and accurately as possible.” This suggests that levodopa may induce a shift from an accuracy-oriented behavior to a speed-oriented one. Interestingly, although patients ON medication were less accurate, their increased movement speed leads to an improved performance as measured by the IP. This suggests that OFF medication the patients' behavior is more goal-oriented whereas ON medication, coordination of both speed and accuracy constraints improves in order to optimize the movement. This finding goes beyond the conclusions of Mazzoni et al. (41) by indicating that dopamine not only “energizes the action” but more importantly optimizes the performance and therefore the energetic cost of the movement.

Finally, it was recently shown that a decrease of beta power in the STN (which is observed after levodopa administration, (42) as well as modulations of C3/C4-STN connectivity is associated with increased speed in a speed-accuracy adjustment task (43). On the other hand, the latter study also found that modulations of low frequency oscillations in a PFC-STN network was associated with increased decision thresholds for accuracy instructions. In our task with constant instruction to move “as fast and accurately as possible,” it is interesting to observe that the behavior changes with levodopa. Whether these two networks are differently recruited, in the absence or presence of levodopa, considering a similar task, remain to be established.

### Structural movement organization

We studied the structural kinematic organization of reciprocal movements using the Hooke portraits and the fitted RD model coefficients. PD patients often have difficulties in maintaining rhythmic movements such as finger tapping, as well as some deficits in timing, i.e., regulation of force and time parameters, rather than simply in force production (19). Advanced PD patients may also demonstrate temporary freezing both of lower and upper limb movements (44, 45). We thus expected difficulties for PD patients in producing the rhythmic aiming task resulting in a shift toward a more discrete pattern of kinematics organization.

In contrast to this hypothesis, our results indicate that PD patients did not adopt a structurally different functional organization than controls regardless their medication state. For both groups, and in line with previous observations, a linear Hooke portrait emerged for low levels of difficulty whereas the nonlinearity increased gradually with increasing accuracy requirements (cf. (10, 45)) (Figures 3, 4). These kinematic adjustments to task difficulty testified of a unique underlying oscillatory dynamics across all IDs tested (10, 46). Moreover, the adaptation of speed of execution after dopamine administration did not lead to a structural movement reorganization.

Some differences between PD patients and the control group were observed in the Hooke portraits, however. Indeed, PD patients' deviations from the mean kinematics patterns were higher than in the control participants (Figures 3, 4) as less variance was captured by the regressions of the trajectories in the Hooke's planes in the PD patients than in the healthy controls. This may be explained in terms of the PD patients' inability to generate appropriate muscle activity that propels the limb to the vicinity of the target (31, 33, 47). Analysis of electromyographical data could be of a particular interest on future works.

However, no differences were found for the four coefficients' increments with ID neither on the acceleration duration between the PD patients and the controls and those for both ON and OFF-medication. The slower PD patients' movement was not due to their movements being performed in a more discrete fashion, but rather suggests a “mere” difference in “time scale” of operation.

As the targets in the present task can be considered as external cues helping patients to keep moving continuously, the movements executed during this task can be seen as externally-guided but internally paced. Motor impairments are observed when PD patients rely on internal control processes but have preserved externally cued performances (48–51). The use of external sensory cues as a strategy to facilitate movement continuity has been extensively documented in the locomotor activity domain using rhythmic auditory stimulation and visual stimulation (52–57). Dynamic visual cues are also involved in the visual perception of one owns actions (58) in terms of optic flow. As PD patients remained seated during the current study, the optic flow cannot be considered as a factor in this study. However, cursor movement relative to the targets in the task space clearly defines as visual information that influences the pattern of motion in the effector space (16). These dynamical visual cues may have helped PD patients to perform the continuous aiming task. Further investigations are warranted to determine whether the kinematic organization of rhythmic movements remains similar in a task where no such visual cues are provided.

Rhythmic movements are known to activate a variety of unilateral primary motor areas (M1, S1, caudal part of the dorsal premotor cortex, caudal portion of the supplementary motor area, rostral portion of the supplementary motor area, caudal cingulate zone, rostral cingulate zone posterior and cerebellum) (7). The lateral premotor cortex (PMC) has been associated with controlling externally-cued visuomotor actions by mapping cues to the appropriate movement (59, 60) and increased connectivity between prefrontal cortex and PMC has been observed in PD patients performing externally-guided tasks (61). This increased connectivity has been considered as a compensatory mechanism, which allows to maintain normal performance during externally-cued movements OFF levodopa (61). Timing in rhythmic movements is also an important parameter. There is evidence that PD patients have impairments in timing processes (30, 62–64) and that movement variability is caused by low frequency activities in the basal ganglia (65) and reduced by high frequency stimulation of the subthalamic nucleus (66). However, the above-mentioned studies used a simple finger-tapping task and had no constraints on accuracy. Further studies are warranted to examine the networks involved in our task and the effects of levodopa thereon.

## Conclusion

This study has investigated how PD affects motor performance in the context of rhythmic goal-directed movements. For the first time, a reciprocal aiming task has been proposed, not only to determine macroscopic parameters of the movements (i.e., speed or accuracy), but also to assess the neuro-motor system's structural organization in PD and how it changes with increasing task accuracy requirements and/or with the DRT. The current study demonstrates that PD patients were able to perform rhythmic and accurate movements. Speed-accuracy trade-off was at work even while PD patients were OFF-medication. Overall, PD patients moved slower than control participants and were less accurate particularly in the OFF-dopamine condition. When it comes to PD patients' structural movement organization, PD does not affect how the neuro-motor system assembles a movement organization in the production of rhythmic aiming movements.

Putting this studies' findings in perspective, it will be of particular interest to study which networks are activated during reciprocal aiming tasks as well as the impact of deep brain stimulation on such movements.

## Author contributions

LF, RH, JI, J-PA, and AE contributed to the conception of the study. LF and AE helped with recruitment and screening of subjects. LF carried out the experiment and performed the data analysis. LF, RH, JI, and AE wrote and/or reviewed the manuscript. J-PA approved submission. All persons designated as authors qualify for authorship, and all those who qualify for authorship are listed.

### Conflict of interest statement

The authors declare that the research was conducted in the absence of any commercial or financial relationships that could be construed as a potential conflict of interest.
